# Performances of five risk algorithms in predicting cardiovascular events in patients with Psoriatic Arthritis: An Italian bicentric study

**DOI:** 10.1371/journal.pone.0205506

**Published:** 2018-10-11

**Authors:** Luca Navarini, Domenico Paolo Emanuele Margiotta, Francesco Caso, Damiano Currado, Marco Tasso, Silvia Angeletti, Massimo Ciccozzi, Raffaele Scarpa, Antonella Afeltra, Luisa Costa

**Affiliations:** 1 Unit of Allergology, Cinical Immunology and Rheumatology, Università Campus Bio-Medico di Roma, Rome, Italy; 2 Rheumatology Unit, Department of Clinical Medicine and Surgery, School of Medicine, University of Neaples Federico II, Neaples, Italy; 3 Unit of Clinical Laboratory Science, Università Campus Bio-Medico di Roma, Rome, Italy; University of Messina, ITALY

## Abstract

**Introduction:**

In patients with psoriatic arthritis (PsA) an increased cardiovascular (CV) risk has been observed. Recently, a EULAR taskforce suggested to use a multiplication by the factor of 1.5 of CV risk algorithms in patients with inflammatory arthritis. This study aims to evaluate the performance of five original and adapted according to EULAR recommendations CV risk algorithms in PsA: SCORE, CUORE, Framingham Risk Score (FRS), QRISK2, and Reynold’s Risk Score (RRS).

**Methods:**

Prospectively collected data from two Italian cohorts were used. Discriminatory ability for CV risk prediction was evaluated by the area under the ROC curves. Calibration between predicted and observed events was assessed by Hosmer-Lemeshow (HL) tests. Sensibility and specificity were calculated for low-to-intermediate and intermediate-to-high risk cut-offs.

**Results:**

One hundred fifty-five patients were enrolled with an observation of 1550 patient/years. Area under the ROC were 0.7679 (95% CI 0.64768 to 0.88812), 0.864 (95% CI 0.79675 to 0.93278), 0.7575 (95% CI 0.65784 to 0.85708), 0.8660 (95% CI 0.79428 to 0.93772), and 0.7183 (95% CI 0.57795 to 0.85862) for SCORE, CUORE, FRS, QRSIK2, and RRS, respectively. HL tests demonstrated poor model fit (p<0.05) for SCORE, CUORE, and RRS. Discriminative ability and calibration were not improved by adaption of the algorithms according to EULAR recommendations. Up to 80% of CV events occurred in patients at “low risk” and up to 93% of CV events in patients at “low-intermediate risk”.

**Conclusions:**

Adaption of the CV risk algorithms according to EULAR indications did not provide improvement in discriminative ability and calibration in patients with PsA.

## Introduction

Psoriatic arthritis (PsA) is an inflammatory arthritis associated with personal or familial history of psoriasis. PsA is characterized by heterogeneous clinical presentation, with possible axial and/or peripheral joint involvement, enthesitis and dactylitis [1].

In PsA, as well as in other forms of inflammatory arthritis, an increased cardiovascular risk (CV) has been observed. Increased prevalence of traditional CV risk factors, chronic inflammation and potential adverse effects of drugs, especially corticosteroids and nonsteroidal anti-inflammatory drugs (NSAIDs), contribute to CV comorbidity in PsA [2–4]. Notably, chronic inflammation can affect global CV risk on multiple points of view. The expression of a more atherogenic lipid profile, the forced reduction of physical activity, and the increased production of cytokines involved in plaque formation, such as interleukin (IL)-1, IL-17, and tumor necrosis factor (TNF)-alpha, seem to boost CV risk of patients [2]. Accelerated atherosclerosis seems to play a pivotal role predisposing patients with PsA to the development of CV and cerebrovascular events [5].

Therefore, the identification of high CV risk patients with psoriatic arthritis is particularly important, in order to implement preventive strategies, like lifestyle changes and pharmacological interventions. A large number of CV risk algorithms have been proposed over time [6]. The performance of algorithms for calculating CV risk in patients with another inflammatory, like rheumatoid arthritis (RA), is a largely debated subject. In particular, Arts and coworkers [7] assessed the performance of four CV risk algorithms in establishing the risk of fatal and non-fatal CV events in European patients with RA. The results show that different scores (Framingham, SCORE, Reynold’s Risk Score, QRISK2) tend to underestimate CV risk in patients with RA; the risk observed exceeds that predicted; the different scores appear slightly calibrated for RA patients [8]. In addition, Ernst and coauthors demonstrated that the Framingham Risk Score (FRS) underestimates CV risk in patients with psoriatic arthritis [9].

Recently, the European League Against Rheumatism (EULAR) recommended to adapt the general population risk algorithms with a multiplication by the factor of 1.5 in patients with rheumatoid arthritis and other inflammatory arthritis, except for QRISK2 which is characterized by a multiplication factor intrinsic to the algorithm for rheumatoid arthritis [10].

Only a few data evaluate the performance of different algorithms, both original and adapted according to EULAR indication, in predicting the actual CV risk in PsA. In this study, the performance of FRS, Systematic Coronary Risk Evaluation (SCORE), QRISK2, Reynolds risk score (RRS), and the Italian Progetto CUORE individual score is evaluated in a bicentric cohort of patients with psoriatic arthritis from Rome and Neaples (Italy).

## Methods

A retrospective analysis of prospective collected data from PsA cohort of Unit of Immunorheumatology, Università Campus Bio-Medico di Roma, and Unit of Rheumatology, University of Neaples “Federico II”, has been made in November 2017. Patients without a personal history of CV disease (CVD) at baseline (November 2007) were consecutively included in this study. At baseline, all the patients fulfilled the Classification Criteria for Psoriatic Arthritis (CASPAR) [11].

Ethics committee of Università Campus Bio-Medico di Roma approved the study, which complied with the Declaration of Helsinki.

Baseline characteristics extracted from the cohort database: were age (years), gender (male/female), weight (kg), height (cm), CRP (mg/l), disease activity score (DAS) 28-joints, axial joint arthritis (Y/N), peripheral joint arthritis (Y/N), enthesitis (Y/N), dactylitis (Y/N), psoriasis area severity index (PASI), history of inflammatory bowel disease (Y/N), history of uveitis (Y/N), family history of CVD (Y/N), smoking status (Y/N/previous), hypertension (Y/N), systolic blood pressure (mmHg), total cholesterol (mg/dl), high-density-lipoprotein (HDL) cholesterol (mg/dl), use of statins (Y/N), use of antihypertensive medication (Y/N), diabetes mellitus (Y/N), atrial fibrillation (Y/N), chronic kidney disease stage IV-V (Y/N), angina or heart attack in a 1^st^ degree relative <60 years (Y/N).

The primary outcome was the first CV event (fatal and non-fatal), as report by electronic patient files. CV events were considered: sudden cardiac death, coronary artery diseases (CAD) (stable and unstable angina pectoris, myocardial infarction), cerebral vascular accident (CVA), transient ischemic attack (TIA), peripheral artery disease (PAD) and heart failure.

The 10-year general FRS for CVD [12], QRISK2 [13], CUORE [14] and RRS [15, 16] were calculated using already published algorithms. SCORE risk charts for low risk countries were used [17]. The items included in each CV risk algorithm are reported in Table 1. Cut-off values that mark the difference between low-to-intermediate risk and intermediate-to-high risk were 10% and 20% respectively, except for SCORE in which cut-off values were 1% and 5%.

**Table 1:** Items included in SCORE, CUORE, FRS, QRISK2, and RRS (grey bars).

	SCORE	CUORE	FRS	QRISK2	RRS
**Age**					
**Sex**					
**Ethnicity**					
**Systolic blood pressure**					
**Total cholesterol**					
**HDL cholesterol**					
**Smoking status**					
**Diabetes**					
**Antihypertensive treatment**					
**Family history of CV disease**					
**Chronic kidney disease (stage 4 or 5)**					
**Atrial fibrillation**					
**Rheumatoid arthritis**					
**Body mass index**					
**High sensitivity C-reactive protein**					

At baseline, data from medical record were used to calculate individual risk for CV within 10 years for all five algorithms. The discriminatory ability of all five algorithms was evaluated using the area under the receiver operating characteristic (ROC) curve, which is similar to the concordance-statistic (*c*-statistic). Calibration was assessed by comparing the agreement between observed and predicted number of CV events in groups stratified in deciles, sextiles, or septiles of the predicted risk, as appropriate, using Hosmer-Lemeshow (HL) test. Fisher’s exact test has been used for analysis of contingency table, while Mann-Whitney test has been used to compare ranks. The sample size, calculated setting a type I error to 0.05, the test power to 0.8, the AUC of the adapted test to 0.7, was 143 patients [18]. All statistical analysis was performed using STATA V.14.

## Results

Data from 155 patients (1550 patient-years) were analyzed. All the patients were Caucasic and lived in Center or South of Italy. During follow-up, 21 patients had a CV event (1.35 events per 100 patient/years): 8 cases of myocardial infarction or unstable angina pectoris, 3 cases of stable angina pectoris, 2 cases of TIA, 4 cases of PAD, 4 cases of HF. No fatal events were reported. The primary outcome was adjusted to fit each CV risk algorithm, leaving 21, 15, 11, 17, and 15 CV events for Framingham, QRISK2, RRS, SCORE, and CUORE, respectively. As the RRS is not applicable to patients with diabetes or younger than 45 years, these patients (n = 58) were excluded, and only 97 patients were included in the analysis of RRS. Patients characteristics are summarized in Table 2.

**Table 2:** Patients’ characteristics at baseline (November 2007).

	Patients with PsA (n = 155)	Patients with PsA without CV event (n = 134)	Patients with PsA with CV event (n = 21)	p value (comparing patients with and without CV event)
Age (years), median (25^th^–75^th^ Pctl)	48 (40–55)	47 (39–53)	56 (49.5–61)	0.0002
Female, n (%)	95 (61.3)	85 (63.4)	10 (47.6)	p = ns
Disease duration (months) at baseline, median (25^th^–75^th^ Pctl)	50.47 (26.1–99.17)	50.47 (26.1–90.02)	62.63 (38.27–105.2)	p = ns
DAS28, median (25^th^–75^th^ Pctl)	3.75 (2.42–4.705)	3.11 (1.65–4.5)	4.33 (3.89–5)	p = 0.01
Axial disease, n (%)	85 (54.83)	73 (54.48)	12 (57.14)	p = ns
Peripheral disease, n (%)	133 (85.81)	115 (85.82)	18 (85.71)	p = ns
Enthesitis, n (%)	80 (51.61)	68 (50.75)	12 (57.14)	p = ns
Dactylitis, n (%)	41 (26.45)	34 (25.37)	7 (33.33)	p = ns
PASI, median (25^th^–75^th^ Pctl)	3.4 (1.575–5.6)	3.2 (1.5–5.6)	3.6 (1.9–8)	p = ns
IBD, n (%)	8 (5.16)	7 (5.22)	1 (4.76)	p = ns
Uveitis, n (%)	14 (9.03)	9 (6.72)	5 (23.80)	p = 0.02
Smokers, n (%)	52 (33.55)	45 (33.58)	7 (33.33)	p = ns
Family history CV, n (%)	85 (54.84)	70 (52.24)	15 (71.43)	p = ns
Atrial fibrillation, n (%)	6 (3.87)	3 (2.24)	3 (14.29)	p = 0.03
Total cholesterol (mg/dl), median (25^th^–75^th^ Pctl)	187 (164–209)	187.5 (168.3–206)	186 (153–217)	p = ns
HDL cholesterol (mg/dl), median (25^th^–75^th^ Pctl)	52 (43–65)	52 (43–64.25)	55 (42.5–72.5)	p = ns
Total cholesterol/HDL cholesterol ratio (n), median (25^th^–75^th^ Pctl)	3.56 (2.80–4.36)	3.58 (2.81–4.50)	3.16 (2.69–4.19)	p = ns
Systolic blood pressure (mmHg), median (25^th^–75^th^ Pctl)	125 (120–135)	120 (120–130)	145 (130–150)	p<0.0001
Antihypertensive treatment, n (%)	50 (32.26)	32 (23.88)	18 (85.71)	p<0.0001
BMI, median (25^th^–75^th^ Pctl)	26.17 (23.67–29.21)	25.93 (23.49–28.73)	27.01 (24.65–30.2)	p = ns
CRP (mg/l), median (25^th^–75^th^ Pctl)	7 (1.6–25)	7 (1.6–25)	5 (0.7–26.5)	p = ns
SCORE, median (25^th^–75^th^ Pctl)	1 (1–3)	1 (0–2)	3 (2–5)	p<0.0001
CUORE, median (25^th^–75^th^ Pctl)	1.8 (0.8–3.6)	1.45 (0.7–2.8)	5.2 (3.4–8.4)	p<0.0001
FRS, median (25^th^–75^th^ Pctl)	2 (0.6–4.9)	1.5 (0.4–3.95)	4.1 (2.9–10)	p<0.0001
RRS, median (25^th^–75^th^ Pctl)	4 (2–6.5)	3 (2–5)	8 (4–15)	p<0.0001
QRisk, median (25^th^–75^th^ Pctl)	4.5 (1.6–9.6)	3.55 (1.375–7.725)	13.4 (10.55–21.75)	p<0.0001

**Fig 1:**
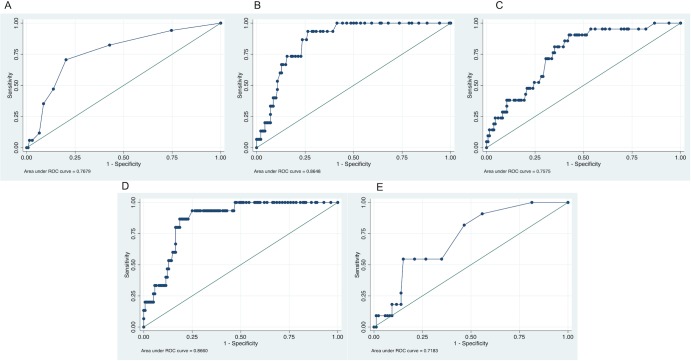
ROC curves for the different original algorithms. Areas under the curve (AUC)-values (95% CI) are 0.7679 (95% CI 0.64768 to 0.88812), 0.864 (95% CI 0.79675 to 0.93278), 0.7575 (95% CI 0.65784 to 0.85708), 0.8660 (95% CI 0.79428 to 0.93772), and 0.7183 (95% CI 0.57795 to 0.85862) for SCORE (A), CUORE (B), FRS (C), QRSIK2 (D), and RRS (E), respectively.

*c*-statistic scores of 0.7679 (95% CI 0.64768 to 0.88812), 0.864 (95% CI 0.79675 to 0.93278), 0.7575 (95% CI 0.65784 to 0.85708), 0.8660 (95% CI 0.79428 to 0.93772), and 0.7183 (95% CI 0.57795 to 0.85862) for SCORE, CUORE, FRS, QRISK2, and RRS respectively were found (Fig 1A–1E).

**Fig 2:**
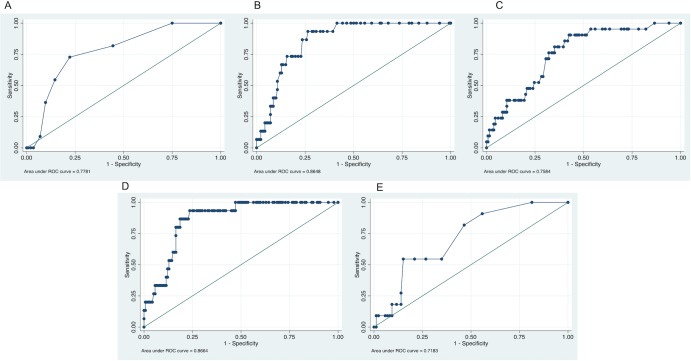
ROC curves for the different algorithms adapted according to EULAR indications. Areas under the curve (AUC)-values (95% CI) are 0.7679 (95% CI 0.64768 to 0.88812), 0.8648 (95% CI 0.79675 to 0.93278), 0.7584 (95% CI 0.65889 to 0.85782), 0.8664 (95% CI 0.79452 to 0.93834), and 0.7183 (95% CI 0.57795 to 0.85862) for SCORE*1.5 (A), CUORE*1.5 (B), FRS*1.5 (C), QRISK2-RA (D), and RRS*1.5 (E), respectively.

Overall, the multiplicative factors do not seems to improve the performances of none of the algorithms: *c*-statistic scores of 0.7679 (95% CI 0.64768 to 0.88812), 0.8648 (95% CI 0.79675 to 0.93278), 0.7584 (95% CI 0.65889 to 0.85782), 0.8664 (95% CI 0.79452 to 0.93834), and 0.7183 (95% CI 0.57795 to 0.85862) for SCORE*1.5 (p = ns vs SCORE), CUORE*1.5 (p = ns vs CUORE), FRS*1.5 (p = ns vs FRS), QRISK2-RA (p = ns vs QRISK2), and RRS*1.5 (p = ns vs RRS) respectively were found (Fig 2A–2E).

**Fig 3:**
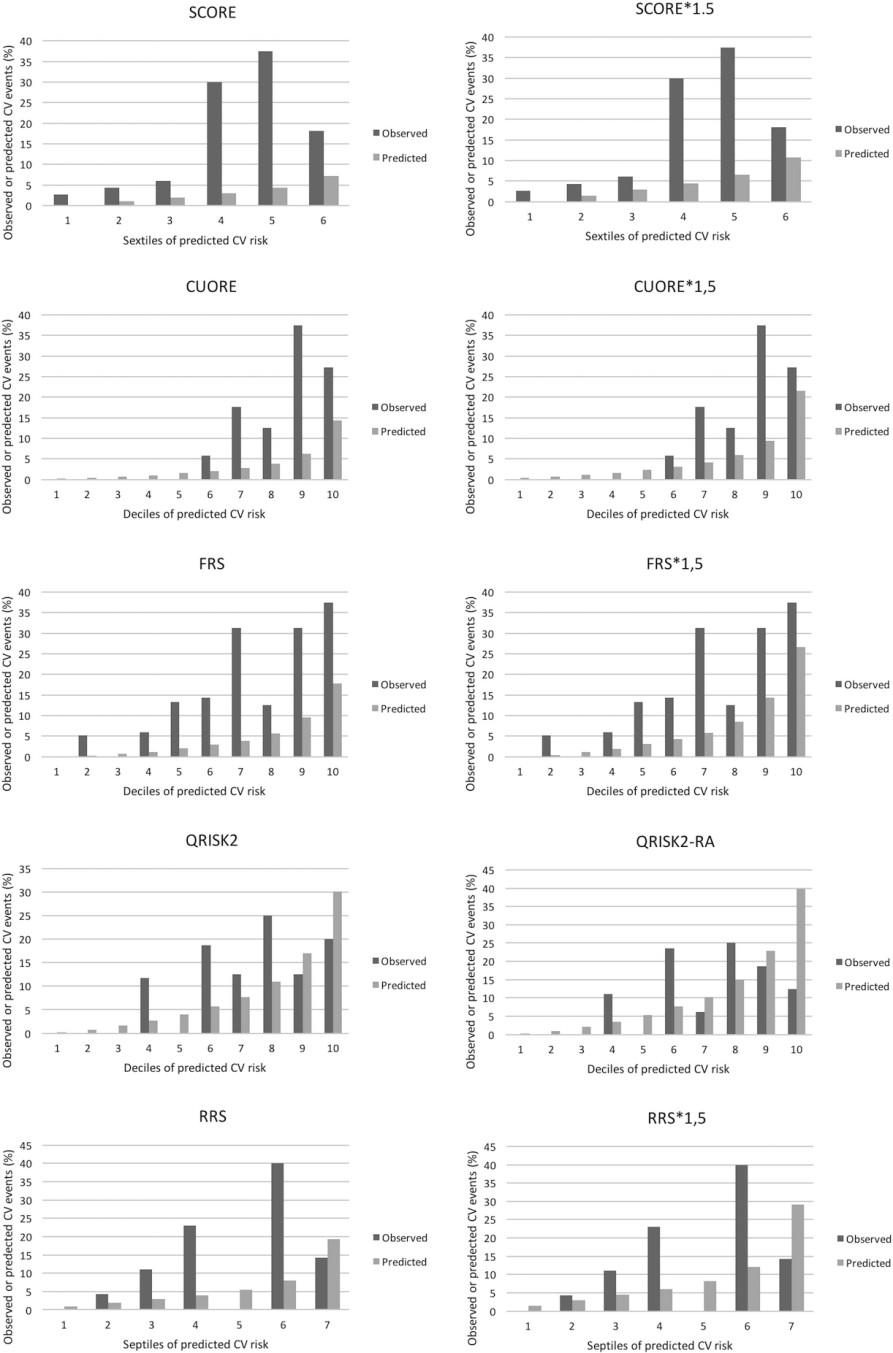
Observed versus predicted risk. Observed (dark grey bars) vs predicted (light grey bars) CV events (%) in deciles, sextiles, and septiles for SCORE (A), SCORE*1.5 (B), CUORE (C), CUORE*1.5 (D), FRS (E), FRS*1.5 (F), QRISK2 (G), QRISK2-RA (H), RRS (I), and RRS*1.5 (L).

Overall, a discrepancy between predicted risk and observed CV events was found (Fig 3A–3E).

CUORE score underestimated CV risk in middle and top deciles and HL test demonstrated poor model fit (p = 0.045); the multiplication by the factor of 1.5 did not improve the performance (HL test p = 0.045). QRISK2 and QRISK2-RA underestimated CV risk in middle deciles and overestimated CV risk in top deciles, and HL test did not demonstrate poor model fit (p = 0.162). CV risk predicted by FRS and FRS*1.5 was lower than observed CV events in every decile, but HL test did not yield a poor model fit (p = 0.26 for FRS and p = 0.259 for FRS*1.5). SCORE and SCORE*1.5 tended to underestimate CV risk in every sextile considered and HL test provided poor model fit (p<0.5). HL test yield poor model fit also for RRS and RRS*1.5 as well (p<0.05).

Sensitivity and specificity of the 10% and 20% cut-off points for CV risk for FRS, QRISK2, CUORE, and RRS and of the 1% and 5% cut-off points for CV risk for SCORE are reported in Table 3.

**Table 3:** Sensitivity, specificity, and OR of cut-off values in CV risk scores.

	True cases (n)	Pos tests (n)	True pos (n)	False pos (n)	False neg (n)	True neg (n)	Tot (n)	Sens (%)	Spec (%)	PPV (%)	NPV (%)	OR	95% CI	p
SCORE >1%	17	73	14	59	3	79	155	82.4	57.2	19.2	96.3	6.2486	1.717 to 22.74	0.0054
SCORE >5%	17	11	2	9	9	135	155	18.2	93.8	18.2	93.8	5.4576	1.4956 to 19.9157	0.0102
CUORE >10%	15	9	3	6	12	134	155	20	95.7	33.3	91.8	5.5673	1.9126 to 16.205	0.0016
CUORE >20%	15	2	1	1	14	129	155	6.67	99.2	50	90.2	9.2143	0.5458 to 155.5474	0.1235
FRS >10%	21	15	5	10	16	124	155	23.8	92.5	33.3	88.6	3.8750	1.1752 to 12.7774	0.026
FRS >20%	21	2	1	1	20	133	155	4.8	99.3	54.5	93.8	6.65	0.3998 to 110.615	0.1866
QRisk2 >10%	15	36	12	24	3	116	155	80	82.9	33.3	97.5	19.3333	5.0657 to 73.7866	<0.0001
QRisk2 >20%	15	11	6	5	9	135	155	40	96.4	54.5	93.7	18.0000	4.6957 to 70.5001	<0.0001
RRS >10%	11	14	2	12	9	74	97	18.2	86	14.3	89.2	1.3704	0.2634 to 7.1294	0.7081
RRS >20%	11	7	1	6	10	80	97	9.1	93	14.3	88.9	1.3333	0.1453 to 12.2368	0.7992
SCORE*1.5 >1%	17	119	16	103	1	35	155	94.1	25.4	13.4	97.2	5.4369	0.6955 to 42.5042	0.1066
SCORE*1.5 >5%	17	27	8	19	9	119	155	47.1	86.2	29.6	93	5.5673	1.9126 to 16.205	0.0016
CUORE*1.5 >10%	15	16	5	11	10	129	155	33.3	92.1	31.3	92.8	5.8636	1.701 to 20.2126	0.0051
CUORE*1.5 >20%	15	6	2	4	13	136	155	13.3	97.1	33.3	91.3	5.2308	0.8731 to 31.3369	0.0701
FRS*1.5 >10%	21	27	8	19	13	115	155	38.1	85.2	29.6	89.8	3.7247	1.3625 to 10.1820	0.0104
FRS*1.5 >20%	21	7	3	4	18	130	155	14.3	97	42.9	87.8	5.4167	1.1201 to 26.1938	0.0356
QRisk-II-RA >10%	15	50	8	42	7	98	155	53.3	70	16	93.3	2.6667	0.9084 to 7.8280	0.0742
QRisk-II-RA >20%	15	20	3	17	12	123	155	20	87.9	15	91.1	1.8088	0.4628 to 7.069	0.3941
RRS*1.5 >10%	11	24	6	18	5	68	97	54.5	79.1	25	93.2	4.5333	1.241 to 16.606	0.0222
RRS*1.5 >20%	11	10	2	8	9	78	97	18.2	90.7	20	89.7	2.1667	0.3973 to 11.8152	0.3716

Abbreviations: Pos: positive; Neg: negative; Tot: total; Sens: sensitivity; Spec: specificity; PPV: positive predictive value; NPV: negative predictive value; OR: odds ratio; CI: confidence interval.

Among patients with CV event, 17.6% was at “low risk” and 52.9% at “low-intermediate risk” according to SCORE, 80% was at “low risk” and 93.3% was at “low-intermediate risk” according to CUORE, 76.2% was at “low risk” and 95.2% was at “low-intermediate risk” according to FRS, 20% was at “low risk” and 60% was at “low-intermediate risk” according to QRISK2, 81.8% was at “low risk” and 90.9% was at “low-intermediate risk” according to RRS, 5.9% was at “low risk” and 52.9% was at “low-intermediate risk” according to SCORE*1.5, 66.7% was at “low risk” and 86.7% was at “low-intermediate risk” according to CUORE*1.5, 61.9% was at “low risk” and 85.7% was at “low-intermediate risk” according to FRS*1.5, 46.7% was at “low risk” and 80% was at “low-intermediate risk” according to QRISK2-RA, and 45.4% was at “low risk” and 81.8% was at “low-intermediate risk” according to RRS*1.5.

## Discussion

The prevention of CV events in general population still remains a challenge. In patients with inflammatory arthritis, an increased burden of CV comorbidity and mortality is well established [10]. Despite in psoriatic arthritis an increased CV risk has been demonstrated [19], little is known about CV risk algorithm in these patients. Overall, the five algorithms evaluated in this study underestimated CV risk. A relatively good discrimination between patients with or without CV events has been demonstrated, with areas under the ROC curves between 0.7183 for RSS and 0.8660 for QRISK2, and calibration of all five algorithms was poor to moderate. In 2015/2016, EULAR suggested to adapt CV risk algorithms by a factor of 1.5 (or to include the independent risk factor RA in QRISK2) in all the patients with RA and virtually other inflammatory arthritis, such as PsA. Notably, in this study the adaptation suggested by EULAR did not increase the discriminative ability and calibration of none of the five algorithms under consideration. Despite the discriminative ability in patients with PsA is comparable to general population, the five algorithms performed less well in terms of calibration [12–17]. Particularly, a poor model fit with a significant different distribution of observed events compared to predicted ones has been demonstrated for CUORE, SCORE, and RSS, but not for FRS and QRISK2. Most of CV events reported in this study occurred in patients at “low risk” or at “low-intermediate risk”. In general population and in patients with PsA as well, people at “low risk” or “low-intermediate risk” are less likely to receive any preventive treatment. Consequently, according to the results of our study, it seems crucial to redefine cut-off values for low and intermediate CV risk in patients PsA. Data reported in the present study demonstrate that, among all the evaluated models, QRISK2 and QRISK2-RA show the best discriminative ability and calibration in Caucasic patients with PsA from Center and South of Italy. These results are in line with other studies. In 2014 Eder and coworkers highlighted that the FRS can underestimate the extent of subclinical atherosclerosis in patients with psoriasis and PsA [20]. Moreover, Shen and coworkers tested the performance of several CV risk scores, including FRS, QRISK2, SCORE, 10-year atherosclerotic cardiovascular disease risk algorithm (ASCVD) from the American College of Cardiology and the American Heart Association, and the EULAR-recommended modified versions by 1.5 multiplication factor in discriminating subclinical atherosclerosis in PsA patients. Also in this case, results showed that all CV risk scores underestimated the subclinical atherosclerosis risk. Further, EULAR-recommended modification improved the sensitivity of FRS and ASCVD only to a moderate level [21]. In a recent cross-sectional descriptive study, Martinez-Vidal and coworkers analyzed the cardiovascular risk according to SCORE charts in 102 PsA patients and studied the presence of subclinical CV disease by common carotid ultrasound. SCORE underestimated the presence of subclinical CV disease in PsA patients. Indeed, according to SCORE charts, above 71% of the patients showed “intermediate risk”, 25.5% “high risk” and 4% “very high risk”. After the ultrasound evaluation, 26.5% of patients were reclassified at “very high risk” due to the presence of atherosclerotic plaques [22].

Several strengths and weakness of this study should be considered. Only 62.6% of patients were eligible for RRS calculation. A comparison between patients with PsA and age-matched and gender-matched healthy controls has not been made. Furthermore, these results were obtained from a population of Caucasic patients with PsA from Center and South of Italy and thus may not be generalizable to patients with other ethnicity or from other parts of the world. Moreover, HL test for QRISK2 and FRS yielded a p value >0.05, suggesting a good model fit, but it cannot be excluded a low power of the test for these algorithms probably due to small sample size.

In conclusion, this study extensively studied five CV risk algorithms in an Italian population of patients with PsA. Overall, these algorithms and their cut-off for low and intermediate risk tend to provide less accurate prediction of CV risk in patients with PsA compared to general population. Underestimation of CV risk in PsA patients could lead to insufficient treatment of CV risk factors. Prospective and larger studies are required in order to improve CV risk prediction in PsA patients, redefining cut-off values of already available algorithms, adding more biomarkers and disease-related CV risk factors in prediction models, and providing PsA-specific CV risk algorithms.
